# Design & development of adulteration detection system by fumigation method & machine learning techniques

**DOI:** 10.1038/s41598-024-64025-4

**Published:** 2024-10-25

**Authors:** Urvashi Agrawal, Narendra Bawane, Najah Alsubaie, Mohammed S. Alqahtani, Mohamed Abbas, Ben Othman Soufiene

**Affiliations:** 1 Department of Electronics and Telecommunication Engineering, Jhulelal Institute of Technology, Nagpur, India; 2 Department of Computer Sciences, College of Computer and Information Sciences, Princess Nourah bint Abdulrahman University, P.O. Box 84428, 11671 Riyadh, Saudi Arabia; 3 Radiological Sciences Department, College of Applied Medical Sciences, King Khalid University, 61421 Abha, Saudi Arabia; 4 Space Research Centre, BioImaging Unit, University of Leicester, Michael Atiyah Building, Leicester, LE1 7RH UK; 5 Electrical Engineering Department, College of Engineering, King Khalid University, 61421 Abha, Saudi Arabia; 6 Prince Laboratory Research, ISITcom, University of Sousse, Hammam Sousse, Tunisia

**Keywords:** Oil Adulteration, Edible Vegetable oils, Random Forest, Sensors, XGBOOST, CATBOOST, Health care, Health occupations, Engineering

## Abstract

A novel method for discovery of adulteration in edible oil is proposed based on concept of refractive index and electronic sensors. The research work focusses on two distinct methodologies like employing datasets and implementing a fumigation technique that integrates real-time hardware for testing Edible oil Impurities. In the first method, the dataset taken into consideration contains spectral data collected using Advanced ATR-MIR Spectroscopy for pure oil and various levels of adulteration with Vegetable oil. Each and every edible oil has a certain value of refractive index. When such oils are contemned in a change adding adulterants, the value of its refractive indices also changes. This value of refractive index serves as a feature for testing the oil and helps us in detecting the adulteration. If Oil is adulterated with vegetable oils, the refractive index will be lower and with animal fats, the refractive index will be higher than that of pure Oil. While in Fumigation Method a hardware module is develop in which adulterated & pure oil samples are heated at 40–50 °C for 4.66 min and the volatiles that are generated by varying gas concentrations are forcefully passed through to the MEMS Gas Sensor-MISC-2714 and Multichannel Gas sensor. The conductance of the sensors changes according to the gases sensed by the sensors contributes to features extraction. The conductance value serves as a feature for the classifier to determine whether the sample is highly, moderately, or lowly contaminated. Thus, in proposed methods we use different algorithms based on machine learning like KNN, Random Forest, CATBOOST and XGBOOST to accurately reveal the adulteration. Amongst all the applied algorithm Random Forest (RF) Classifier & XGBOOST algorithm outperform well and gives 100% accuracy. The proposed work is used for identifying food adulteration in edible food products which helps us to feed Society with high-quality food.

## Introduction

Food adulteration refers to the addition of inferior or harmful ingredients to food products in order to raise the quantity or make it appear more attractive or desirable. The adulterants used reduce the eminence of foodstuff by decreasing its nourishment value as well as by removing or substituting the key ingredients. Food can be tainted at any point in the supply chain, from production to retail. It can be intentional, with the goal of increasing profits for the seller, or accidental, due to contamination or other factors. Some instances of foodstuff adulteration include adding water to milk, mixing sawdust with flour, or adding color to spices to make them appear fresher with higher quality^1,2^. Adulteration can also involve adding harmful substances such as chemicals, pesticides, or heavy metals, which can pose serious health risks to consumers. Food adulteration is a major problem because it compromises consumers’ faith in the safety of their food sources and can cause serious health consequences^3,4^.

Food adulteration is a hot topic due to its significant implications for health, trust, and economics. Several examples marketed as pure honey are actually blended with sugar syrups made from plants including corn, rice and sugar cane, according to numerous research and investigations on honey adulteration. According to recent studies, example of several goods with the designation extra virgin olive oil were really diluted with less expensive oils, such as soybean or sunflower^4^. A number of complaints concerning the rampant adulteration of milk with water, detergent, urea, and other hazardous substances to extend its shelf life and volume surfaced from India in 2023. According to a study conducted in 2022, a substantial portion of spices, including paprika, saffron, and turmeric, were tampered with using dangerous fillers and fake colours, such as lead chromate, in order to improve their colour and weight. Recent DNA testing has shown that a significant amount of seafood served in markets and dining establishments is mislabeled, with less expensive fish being marketed as premium types. For example, tilapia has been sold and labelled as red snapper in the United States^2^. Thus, in the area of regulatory monitoring and industry alertness, major contribution of adulteration detection is involved in food products like honey, olive oil, milk, spices and seafood^5^.

Food adulteration detection is highly relevant to the food industry for several reasons like consumer health and safety, maintaining consumer trust, market competitiveness, technological advancements, maintain sustainability & ethical standards as well as health initiatives. In order to protect consumer health, uphold brand reputation and trust, adhere to regulatory requirements, remain competitive in the market, and support ethical and sustainable food production methods, food adulteration detection is essential in food industry. It promotes public health campaigns and upholds the general integrity and calibre of the food industry. Ensuring the safety and integrity of food items remains a top priority as the food business develops.

**Figure 1:**
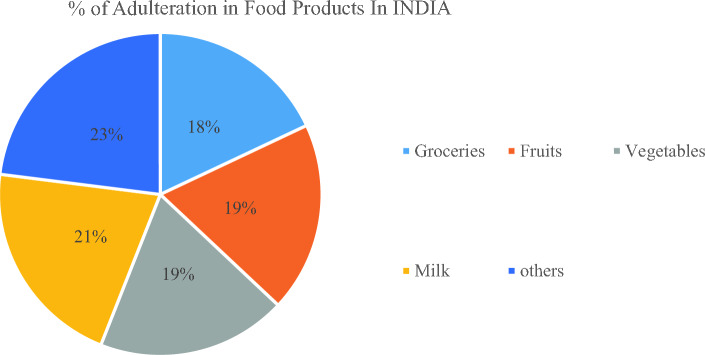
Percentage of Adulteration in Food Products.

To prevent the spread of adulteration and to guarantee safety and authenticity of food supply, governments and regulatory agencies all over the world have enacted rules and regulations, still it does not stop the food adulteration. The Fig. 1 shows the % of adulteration in food product with the help of Pie chart. In the proposed work the food product viz. oil is selected for identification of adulteration.

Oil adulteration denotes the practice of accumulating ingredients to oil that are not meant to be part of the product, in order to increase its quantity, alter its texture or appearance, or reduce its cost. In an effort to sell more product at a lower price, some suppliers may adulterate oil with less expensive vegetable oils like coconut oil or palm oil some common types of oil adulteration include^5–7^.

### Adding artificial colors

Some sellers may add artificial colors to oil to make it look more attractive or to hide any impurities.

### Adding starch or other thickeners

Adding starch or other thickeners to oil can alter its texture and make it appear thicker, creamier, or smoother.

### Adding animal fats

Some sellers may add animal fats such as lard or tallow to oil in order to increase its quantity or to make it cheaper to produce^8,9^.

### Adding preservatives

Adding preservatives such as sodium benzoate or formaldehyde can increase the shelf life of oil but can also be harmful to health.

Oil Adulteration is illegal and can be dangerous for consumers, as it can lead to health problems^10^. To ensure that you are buying pure and unadulterated oil, it's important to purchase it from reputable sources and to check the label for any added ingredients. There are several methods to detect oil adulteration. Some of the commonly used methods are:

### Chemical tests

In oil chemical tests are used to identify occurrence of adulterants. One such test is the Vanaspati Test, which involves adding a small amount of hydrochloric acid to the sample. If the sample turns pink, it indicates the presence of vegetable oil. Similarly, the Adulterants in oil test uses a combination of chemicals to detect the presence of adulterants^11,12^.

### Physical tests

Physical tests are used to spot variations in the corporeal properties of oil, such as its melting point, refractive index, or specific gravity. These tests are profound to changes in the composition of the illustration and can indicate the presence of adulterants.

### Chromatography tests

Chromatography tests are used to separate and identify the various components of oil. In Chromatography tests, the adulterants are detected using High Performance liquid chromatography and gas chromatography, can be used to show the presence of adulterants^13^.

### DNA testing

DNA testing allows for the discovery of animal fats in oil. This experiment is based on the fact that different animal fats have different DNA sequences, which can be identified using genetic testing techniques^14^.

### Sensory analysis

Sensory analysis involves evaluating the taste, smell, and appearance of oil. Any changes in the sensory properties of the oil can indicate that the adulterants are present.

It's important to note that some of these methods require specialized equipment and expertise and may not be accessible to the general public. However, consumers can still protect themselves by purchasing oil from reputable sources and checking the label for any added ingredients^15^.

## Materials and methods

In the proposed work Spectrophotometric analysis is utilize to identify the contamination in olive oil with different oils like sunflower, maize and soyabean oils. The methods proposed by the author are Electrospray ionization mass spectrometry (ESI–MS) fingerprinting analysis and Gas chromatography mass spectrometry (GC/MS). Both methods help to find purity of oils by distinguishing it from other edible oils. The multivariate discriminant analysis classification technique is used to label the pure or contaminated sample of virgin coconut oil with a threshold of 1% adulteration^16^. The other advanced approaches like fatty acid profiles can also be used for further studies by taking into account the geographical and seasonal differences^17^.

In recent years numerous incidents were found in which inferior substances have been added to edible oils. This analysis has been done to address the issues of adulteration in edible oils using detection technology and regulatory compliance. Chromatography and Spectroscopy are two examples of many detection technologies available for edible oils^18^. Nowadays the use of electrochemical sensors such as electric nose and tongue has been on the rise for detection purposes. Such detection methods have the potential to fight against food tampering. Intentional addition of adulterants to edible oils can have devastating effects on both the public health and economy. It is necessary either to develop new technology or improve upon existing ones in order to better monitor the food supply chain^19^. Detection of oil adulterants by various analytical methods are available using expensive equipment and highly skilled personnel^20^.

R. Muthukumar et al. proposed microfluidic colorimetric exposure for visible identification of adulteration. The deciphering of data was done by ultraviolet visible spectrometry and digital image analysis. The quality of visual signal captured by the apparatus can be improved by adjusting the phenolphthalein concentration. The sample was tested for six days, and no discernible changes were observed.

Combination of detection gadget with smartphone images helps in on site data collection. The method proposed by the researcher can be used in other portable platforms as well for monitoring and assessing the food quality^21^.

In FMCG food and beverage industry, luxury edible oils like extra virgin olive oil are more prone to economic driven quality degradation or adulteration. The present quality control procedures are intrusive, offline, sluggish and costly which makes it inefficient at detecting adulteration. The author employed capacitive sensing as it is not intrusive^22^. Non-invasive screening of EVOO filled containers can be done by monitoring the changes in dielectric properties of combined oils using capacitive field without sample extraction. It is well suited for high throughput testing with a reaction time of 100 ms^23^.

Development of a magnetic resonance relaxation fingerprinting method due to low field nuclear magnetic resonance spectroscopic investigations of edible vegetable oils is simple, accurate and fast. Zhi-Ming Huang et al.^24^ proves that relaxation spectroscopy helps in type identification of six different edible vegetable oils with their unique fingerprints. Oil adulteration is diagnosed using PCA by filtering out three separate regions in three fingerprints. In the proposed method, the edible oil adulteration is successfully identified and quantified using partial least squares regression and univariate analysis.

Huq et al.^25^ proposed a compilation summary of rapid detection methods to get the general idea about the extent to which the food is adulterated at home as number of methods used to determine the purity of fat or oils are time consuming and laborious. Ghee tainted with vanaspati, and butter dyed yellow are two of the contaminated food examples out of many. Consumer confidence has dropped as they are doubtful regarding the purity of products both at retail and wholesale level due to the deficiency in market survey. In retail settings acid-based alkali-based color-changing rapid detection kits have been formed for easy and quick identification of oils and fats adulteration. Law enforcement organizations should be more attentive to properly administering the law and inform the consumer regarding the adulteration methods so that they can receive pure edible fats and oils. In recent years numerous incidents have been found in which inferior substances are added to edible oils. Choon Hui Tan et al.^26^ put a light on the issues of edible oil adulteration, its detection and regulatory compliance.

Many adulterations detection technologies method based on spectroscopy and chromatography are developed for edible oils. It is necessary to either develop new technology or improve the existing ones in order to better monitor the food supply chain.

Vegetable oil adulteration analytical methodologies that are easy, sensitive, swift, accurate and trustworthy is a growing need for international institutions to draw antifraud legislation to regulate the worldwide trade in oil. Currently regulations for oils are falling within the adequate compositional boundaries so it is necessary to refine current methodologies for describing and analysing petroleum products. O. Abbas et al.^27^ applied new bio molecular approaches progressively to identify vegetable oil contamination over traditionally available chromatographic and spectroscopic techniques.

Essential oil (EO) is adulterated by the insertion of less expensive vegetable oils or synthetic EO. Such manipulation has no effect on qualitative composition, but it requires an absolute quantitative examination for adulteration diagnosis. Francesca Capetti et al.^28^ offered use of two procedures to recognize EO tampering using examples that featured adulterated versions of popular commercial EO as well as EO of different origins and inferior economic values. It shows that given a reference quantitative profile, absolute quantification is required to draw attention to adulteration with fattier vegetable oils.

The oil use patterns, shopping habits and respondents’ demographic information were gathered using a practice and knowledge questionnaire. According to FSSAI guidelines evaluation of the chemical adulterants was conducted. As per survey conducted, people from higher socioeconomic backgrounds showed greater awareness of oil adulteration. A total of 392 samples (39.28% of the unpackaged and 31.25 percent of the packaged) were found to have chemical signs of adulteration^29^.

Anindita Deb Pal et al.^30^ indicated that people with the higher income group had a greater awareness of the issue of oil adulteration than those in the lower income group. The widely used cooking oils like packaged and bulk samples of soyabean and mustard oils revealed presence of adulterants by chemical testing. It proves that even buying sealed packaging oil gives no assurance of its purity. More education needs to be incorporated in the public to make them aware of proper food safety practices.

Iandae et al. proposed an overview of analysis of Coconut oil by integrating spectroscopy with Artificial Intelligence. The analysis is done in terms of detection, quality control and authentication. The result summary shows the available state-of-the-art revolutionary technologies showcasing the ability of spectroscopic technique with AI to improve and transform precision in industry related to coconut oil^31^.

Juliana et al. proposed method for detection of adulteration in raw bovine milk by combining differential scanning calorimetry with machine learning algorithms namely RF, MLP and GBM. DSC Profile of adulterated samples are different from raw milk.

RF, GBM and MLP were capable of predicting 88.5%, 100% and 100% of adulterated samples correctly. The vital discriminating predictor in case of RF, MLP and GBM models is crystallization as well as boiling peak temperature^32^.

A. Menevseoglu et al. proposed NIR spectroscopy for almond adulteration detection which is cheaper than traditional chromatography techniques. The benchtop spectrometer helps in collection of NIR spectra on which analysis is done by combining partial least square regression with conditional entropy and soft independent modeling of class analogy. The NIR spectral data differentiate the pure and adulterated sample clusters with 100% accuracy^33^.

Iuri Lima dos et al. describes that in underdeveloped countries Yoghurt and other milk derivatives are seriously threatened by the pervasive and economically motivated practice of adulterating milk with water. The Author examined the effects of adding 0–15% water to milk on plain yoghurt quality characteristics over 28 days of refrigerated storage, even though there are no required water detection tests for plain yoghurt. Colour and texture were significantly impacted by starter culture dynamics. However, sensory analysis showed little effect on how consumers perceived it. These findings highlight the possibility of inadvertently ingesting contaminated yoghurt and highlight the need for strict regulatory controls to prevent such practices^34^.

Rafael et al. helps to find the adulteration in grated Italian hard granular cheese called as Parmigiano Reggiano cheese with low value cheeses namely Ricotta. The statistical techniques were employed in conjunction with urea polyacrylamide gel electrophoresis to find out presence of Ricotta in grated Parmigiano Reggiano cheese. Six distinct ratios of grated Parmigiano Reggiano cheese to grated Ricotta cheese were used by author to create the tampered samples. The Naïve Bayes Classifier outperformed rank correlation and logistic regression for cheese samples having more than 20% adulteration with Ricotta in terms of evaluation parameters like accuracy and receiver operating characteristic curve. This research might be viewed as a pilot project to examine how machine learning techniques might be used in forecasting genuineness of Parmigiano Reggiano cheese^35^.

Most of the researchers carried out their work based on chemometric techniques instead of spectroscopic techniques to identify adulteration. The calibration method used in earlier research work is also not reliable and stable. In the proposed work two different approaches are used, one with the available database and other by collecting the real-time database by means of hardware. The dataset serves as the foundation for identifying adulteration in oil with the help of refractive index. While Fumigation method make use of electronic sensors that offers a real world and practical aspect to the research work. Thus, both the proposed research approaches are reliable, stable and indispensable in their own way. The proposed work introduces an innovative way by moving away from the conventional method of chemical analysis with machine learning. As an alternative, it makes use of datasets and electronic sensors in unison with Machine learning to detect adulteration.

The Existing work shows that most of the methods available for detection of adulteration are not useful practically as it is very time consuming, needs costly apparatus, specialized training and do not have adequate benefit practically. To gain research interest and to solve issues present in the existing work, the design methodology is proposed.

## Design methodology

The food product namely oil is taken into consideration for detection of adulteration. The Methodology was proposed in two ways. In the First Section different Machine Learning algorithms are applied on oil dataset. In the second section, we develop a hardware module based on Fumigation method with the help of Multichannel gas sensor to identify the existence of adulteration in oil.

### Detection using dataset

**Figure 2:**
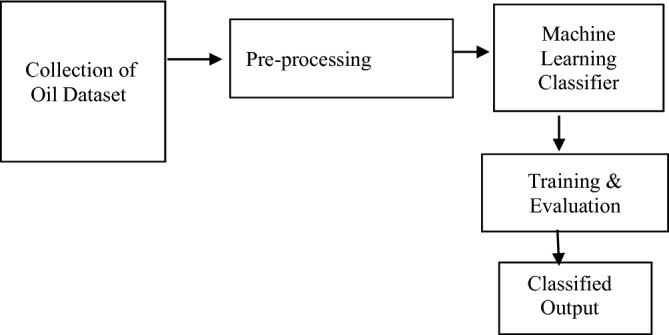
Block diagram for detection using dataset.

The Proposed block diagram for detection method is shown in Fig. 2. The pure as well as adulterated oil samples dataset are used from the available one for the research work. The refractive index value extracted from this dataset is then given to the classifier which will classify the sample to show whether adulteration is high, medium or low.

The amount by which a substance causes light to bend when it travels through it is known as its "refractive index." The refractive index values help to prove that the oil has been tampered with. Different substances have different refractive indices, so deviations in refractive index can indicate existence of adulterants. A substance's ability to bend light is quantified by a refractometer. To detect oil adulteration using refractive index, a refractometer is used. The sample is placed on the prism of the refractometer, and a light is shone through it. The amount of refraction is measured, and the refractive index of the sample is calculated.

If oil is adulterated with vegetable oils, the refractive index will be lower than that of pure oil. This is because vegetable oils have a lower refractive index than oil. Similarly, if oil is adulterated with animal fats, the refractive index will be higher than that of pure oil. The refractive index is just one of the methods that can be utilize to detect oil adulteration, and it is often used in combination with other methods such as chemical tests or sensory analysis to provide more accurate results.

The dataset contains spectral data collected using advanced ATR-MIR Spectroscopy for pure oil and various levels of adulteration with Vegetable oil. The data was collected at 128 wavelengths, and there are a total of 160 data samples, with 40 samples for the pure class and 40 samples for each of the 50%, 25%, and 6.25% adulterated classes. The dataset matrix consists of overall 160 rows × 130 columns in which pure samples, 50% adulterated samples, 25% adulterated samples and 6.25% adulterated samples each consists of 40 rows × 130 columns. The data was collected using state-of-the-art spectroscopic techniques, ensuring high accuracy and reliability. The dataset can be used for classification tasks to identify the existence of vegetable oil contamination in oil. It can be useful for researchers and data scientists working in the food industry, specifically for product authentication and quality control. Machine learning algorithms are applied on the dataset for detecting adulteration in edible oils using similar spectroscopic methods.

Predictive models of Machine learning algorithms are used in developing adulteration detection system based on refractive index of oil determined by its chemical properties. By training a dataset of refractive index values based on chemical properties of various types of oil using machine learning algorithm could give accurate predictions for a given set of inputs. Some of the chemical properties that could be used as inputs for a machine learning model include the free fatty acid content, moistness content, and hydrogen peroxide value of the oil. These properties are known to affect the refractive index of oil, so including them as inputs can improve the accuracy of model.

Once the model is trained, it can be utilized to calculate the refractive index of new samples of oil based on their chemical properties. Thus, adulteration in oil can be detected by distinguishing between pure oil and oil adulterated with other substances. It's crucial to remember that quality of data used for training machine learning models directly affects accuracy of the resulting models. Additionally, other factors such as the presence of impurities or other types of adulterants can affect the refractive index of oil, so machine learning models should be used in combination with other detection methods for best results.

Applying machine learning to a refractive index dataset involves several steps. Preprocess the data to ensure that it is suitable for machine learning. This may involve data preparation steps including normalizing, scaling, and separating the data into test and training sets. Use the training data to teach the machine-learning model. Here, we minimize the discrepancy between the observed and anticipated refractive index values by fitting the model to the training data. Examine how well the model performs on testing data. This involves measuring the accuracy of model predictions and comparing them to actual refractive index values. If the model performance is not satisfactory, optimize the model by adjusting the model architecture or hyper parameters. Once a satisfactory model has been trained, it can be used to predict the refractive index of new samples based on their chemical properties (Table 1).

**Table 1:** Outlines the details of food product use with the respective machine learning methodologies employed so far in Food Science.

Author Name and Ref. No	Food product	Machine learning technique	Remark
Li et al.^36^	Sour Skin of Onions was Detected	SVM & PCA MANOVA for hypothesis testing	Accuracy 81% during training & 85% for validation
Mu et al.^37^	Pure Oil & Mixed Oil	Multivariate Analysis, ANN and SVM	Pure oils are classified well
Rashvand et 1 l.^38^	Mixture of Olive oil, Sunflower oil & Canola oil	Dielectric technique with LDA	Accuracy 72%
Teye et al.^39^	Cocoa Beans	LDA & PCA using E-Nose	Accuracy-76.5%
Ordukaya et al.^40^	Olive Oil	Naive Bayes & KNN	Accuracy- 70.83%
Ayari et al.^41^	Cow Ghee	PCA & ANN	Accuracy PCA- 96% Accuracy ANN- 91.3%
Jhuria et.al.^42^	Apple Disease	ANN	Accuracy 90%

### Detection using fumigation method

**Figure 3:**
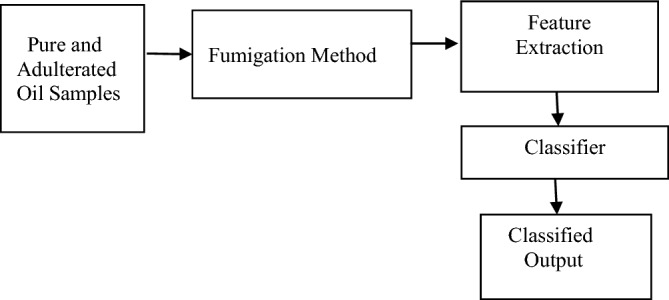
Block diagram for detection using Fumigation Method.

**Figure 4:**
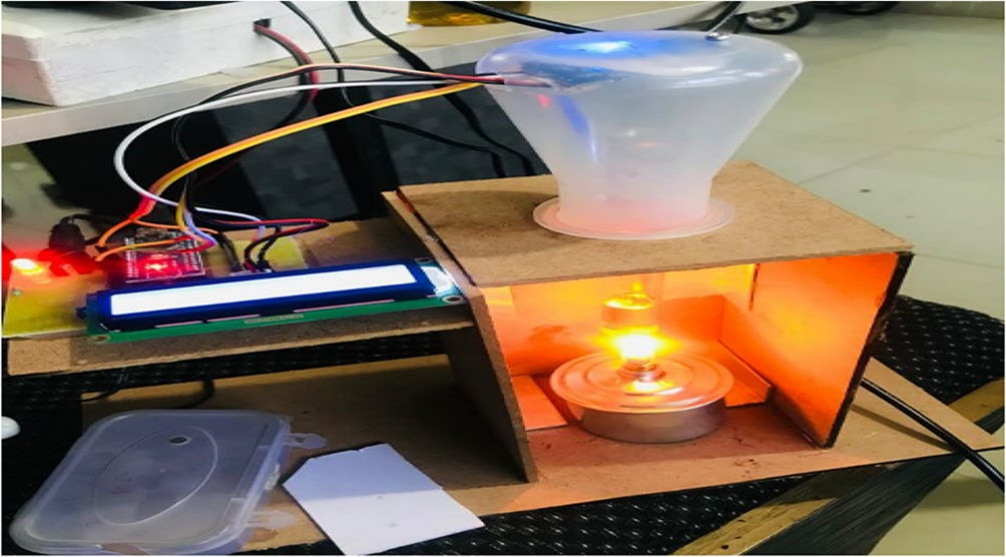
Developed Hardware.

**Figure 5:**
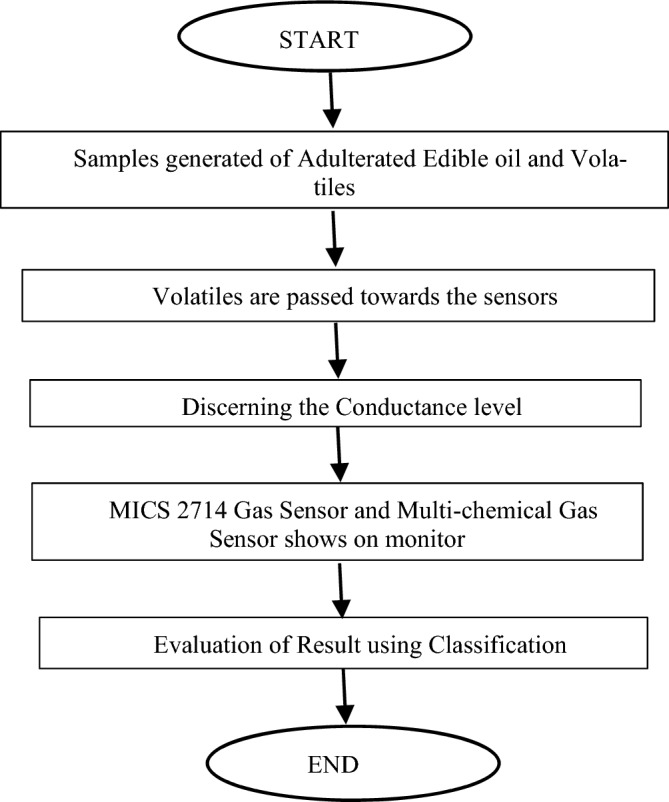
Flowchart for Fumigation Method.

The proposed block diagram for Fumigation method is shown in Fig. 3. The pure and adulterated oil samples are heated, and volatiles are generated from it which is passed through Sensors. The conductance of the sensor changes according to percentage of adulteration. The Feature extracted are then applied to the Machine Learning Classifier to finally detect the output. Figure 4 shows the actual developed hardware. Figure 5 shows the Flowchart for Fumigation Method.

The contaminated samples are made by mixing different volumes of Palm oil with other edible oils. Samples of tainted Edible oil were prepared with total of 5 ml from Palm oil and Edible oil, as described in Table 2 below.

**Table 2:** Percentage Mixing of Edible oil with Palm oil.

Sr. No	Edible oil	Palm oil	Mixing percentage
	4.0 ml	1.0 ml	20%
	2.5 ml	2.5 ml	50%
	1.5 ml	3.5 ml	75%
	5.0 ml	0.0 ml	100%

These samples are heated at 40–50 °C for 4.66 min and the gases of different concentrations that make up the volatiles which are generated are forcefully passed through to the MEMS Gas Sensor-MISC-2714 and Multichannel Gas sensor. Here Arduino is used in addition with an analog extender. Since Arduino consists of only single analog pin. It facilitates five analog signals for two sensors. The serial monitor of Arduino Programme shows data obtained from the sensors. The sensors conductance changes according to the gases sensed by the sensors. Data analysis is done using a classifier. The features collected using the proposed method are given into a classifier, which then determines the level of contamination in the sample.

The dataset collected contains information of volatiles sensed by the multichannel gas sensor for edible oil and various levels of adulteration with palm oil. The data collected contains 280 samples each for each pure as well as 20%,50% and 75% adulterated classes. The dataset matrix consists of 280 rows × 6 columns of pure as well as each 20%, 50% and 75% adulterated samples.

### Machine learning algorithms

The Machine Learning algorithms consist of K Nearest Neighbors, Random Forest Classifier, CATBOOST and XGBOOST. KNN computes the nearest neighbors to predict the output for test class. The Random Forest method produces a set of decision trees, polls all trees for their predictions, and then picks the one with the highest total to predict the output of test class. Catboost is a special case of gradient boosting that works well with both numerical and categorical features and also takes cares of missing values in the dataset. XGBoost involves parallel processing capabilities for training models on datasets in a practical period of time and takes care of missing values without extensive preprocessing. Each algorithm is explained individually in detail in further subsection.

#### K nearest neighbors

The general outline of the steps involved in applying KNN to dataset includes collection of datasets, preprocessing of data, choosing the value of k, training, evaluating, optimizing & using the model to predict the class of adulteration. The value of k is decided, which is the number of nearest neighbors utilized to compute an upcoming sample's refraction index. Eucledian distance is used to calculate the distance D (a,b) between two nearest neighbors a and b which is given by Eq. (1), below:

1 $$D\left(a,b\right)=\sqrt{\sum_{j=1}^{n}{(aj-bj)}^{2}}$$

The optimal value of k depends on characteristics of dataset and can be determined using cross-validation techniques. The given data must be folded, or partitioned, into many subsets before the model can be trained, with one-fold acting as a validation set. This is repeated multiple times, each time using a unique set of folds as the validation set. Then, the overall validation results are averaged to estimate the model's performance. Training data in KNN model involves storing the training data in memory and calculating the distances between the new sample and each of the training samples. The k nearest neighbors is then selected to predict the class of the new sample.

#### Random forest

The Random Forest method produces a set of decision trees, polls all trees for their predictions, and then picks the one with the highest total. The over-fitting is mitigated by averaging the results from multiple decision trees, making the ensemble method superior to a single decision tree. The first step is to pick random samples from the data set. The program then constructs a decision tree tailored to each sample. Then, it will compile the predictions made by each decision tree. In this stage, votes will be taken on every possible conclusion. Last but not least, go with the most popular predicted result. The classification is done by taking help of gini impurity for each label at a node which is calculated by using following Eq. (2).

2 $$Gini Impurity=\sum_{j=1}^{m}gj\left(1-gj\right)$$

where, m represents the number of unique labels or classes in the dataset, j denotes each individual label or class from 1 to m, gj represents the frequency of label j at a specific node in the decision tree.

#### CATBOOST

CatBoost is a supervised machine learning method with two main features namely categorical data (the Cat) and its gradient boosting (the Boost). CatBoost namely "Categorical Boosting," is a boosting library created by Yandex that is freely available to the public. It is intended for use on issues involving a large number of features, such as regression and classification. Catboost is a special case of gradient boosting that works well with both numerical and categorical features. To further mitigate over fitting and boost overall efficiency, it employs an approach called symmetric weighted quantile sketch (SWQS), which takes care of missing values in the dataset automatically. Label encoding in CatBoost algorithm is calculated by the Eq. (3):

3 $${\text{Lable}}\,{\text{Encoding}}\,{ = }\,\frac{{{\text{Current\_value}}\,{\text{(a*prior)}}}}{{{\text{maximum\_value + a}}}}$$

Where and prior are the constant parameters, Maximum value is the sum of all items in the same category as the current row in the dataset, while current value is the sum of all things in same category up to current row.

#### XGBOOST

As an ensemble learning technique, ("Extreme Gradient Boosting") takes the predictions of several very ineffective models and merges them into a single, more accurate one. XGBoost's efficient handling of missing values is a powerful feature that allows it to work with real-world data that contains missing values without requiring extensive pre-processing. In addition, XGBoost's native parallel processing capabilities allow for training models on big data sets in a practical period of time. The Loss function for simplified XGBOOST algorithm is given by Eq. (4).

4 $$L\left(t\right)=\sum_{i=1}^{n}\left[gift\left(xi\right)+\frac{1}{2}hi {ft}^{2}\left(xi\right)\right]+\Omega \left(ft\right)$$

Where, xi represents a positive outcome, n is the trees number, f represents the functional space that contains positive outcomes, and Ω is regularization parameter.

## Result and discussions

Mixing Edible oil and Palm oil in tube on 5 ml and After heating for 4.66 min. It is found that the Multichemical gas sensor and MICS-2714 sensor is more sensitive to the volatiles from the heated samples of the adulterated Edible oil.

The NO2, C3H50H, VOC, CO, H2 level of the adulterated Edible oil samples for Palm oil is increased with the adulteration, while for the Edible oil it decreases. Table 3 shows the reading for different types of gases according to percentage of adulteration.

**Table 3:** Readings for detecting adulteration of Edible and palm Oil.

Proportion of edible and Palm oil	Time	NO2	C3H5OH	VOC	CO	H2
Edible oil (4.0 ml) + palm oil (1.0 ml)	2	2.87	168.11	219.51	309.44	0
Edible oil (2.5 ml) + palm oil (2.5 ml)	2	3.78	119.77	269.2	334	0
Edible oil (1.5 ml) + palm oil (3.5 ml)	2	1.64	150.9	163.39	306.52	0
Pure Edible oil	2	3.54	225.87	304.89	270.32	0

**Figure 6:**
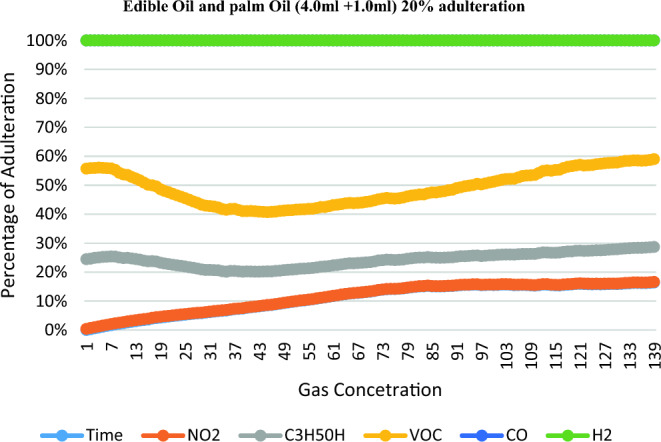
Edible oil and Palm oil (4.0 ml + 1.0 ml) 20% adulteration.

**Figure 7:**
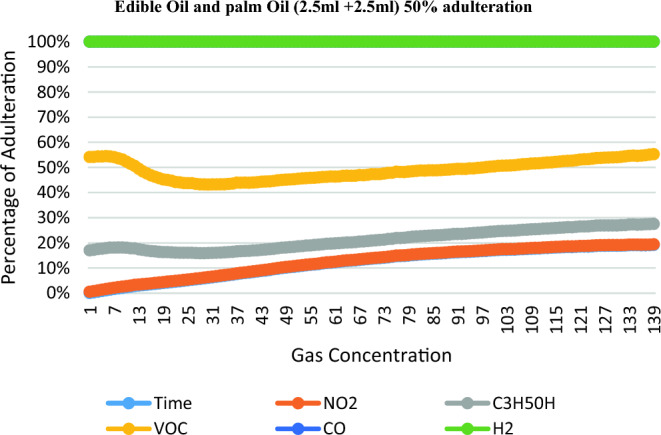
Edible oil and Palm oil (2.5 ml + 2.5 ml) 50% adulteration.

**Figure 8:**
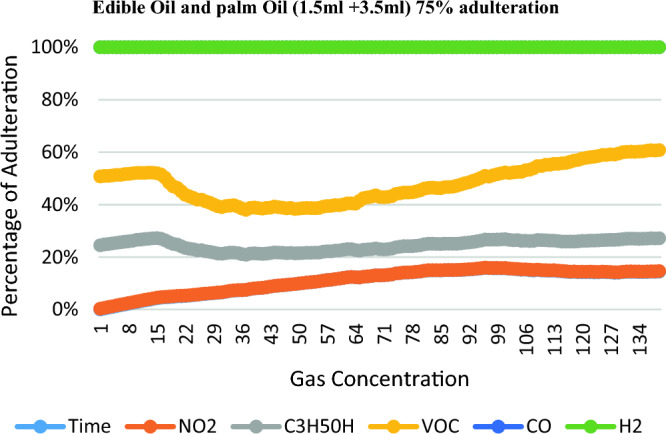
Edible oil and Palm oil (1.5 ml + 3.5 ml) 75% adulteration.

**Figure 9:**
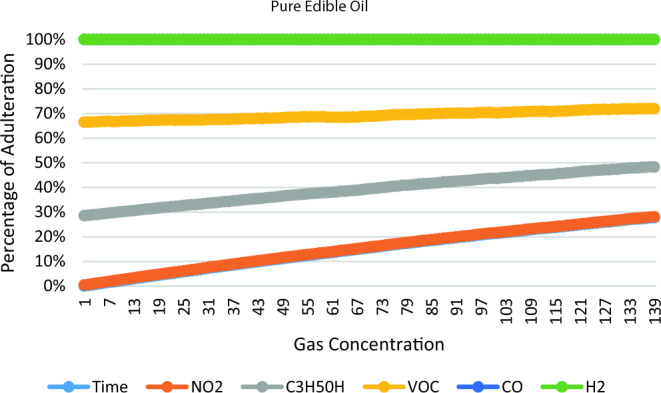
Pure Edible oil.

According to the reading shown in Table 3, Figs. 6,7,8,9 shows the graph depicting the percentage concentration of different gases sensed by the Sensor according to 20%, 50%, 75% adulteration & Pure Sample respectively.

**Figure 10:**
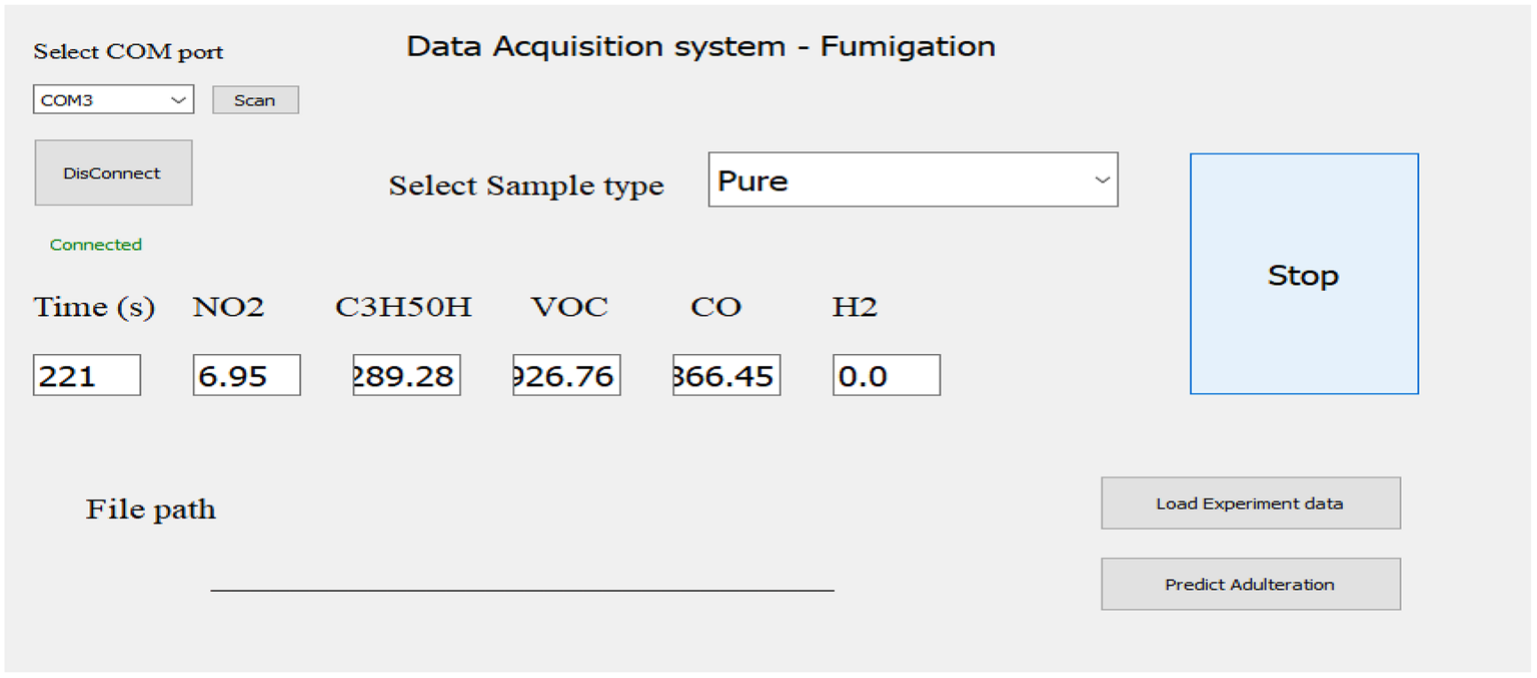
GUI for Fumigation Method.

Figure 10 shows the Graphical user interface for fumigation method. In this GUI concentration of different gases for pure sample with time is being shown.

**Figure 11:**
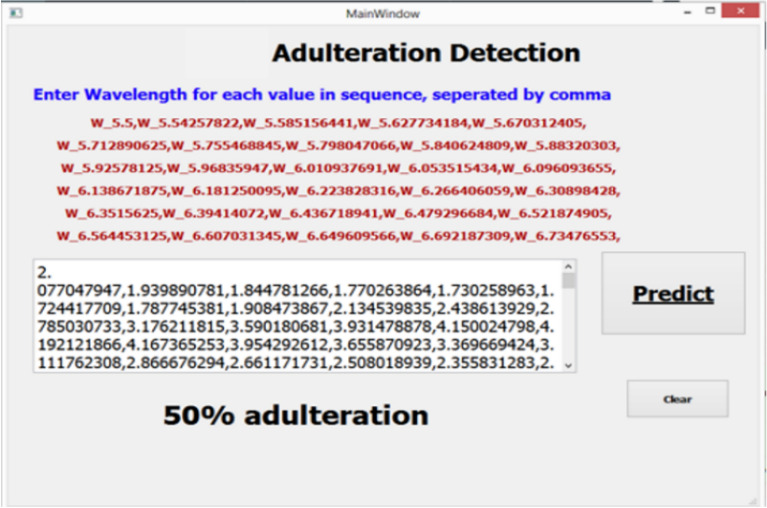
GUI for Detection using Dataset.

Figure  11 shows the Graphical user interface for detection of adulteration using dataset. In this GUI refractive index value by means of wavelength for 50% adulterated sample is being shown.

**Figure 12:**
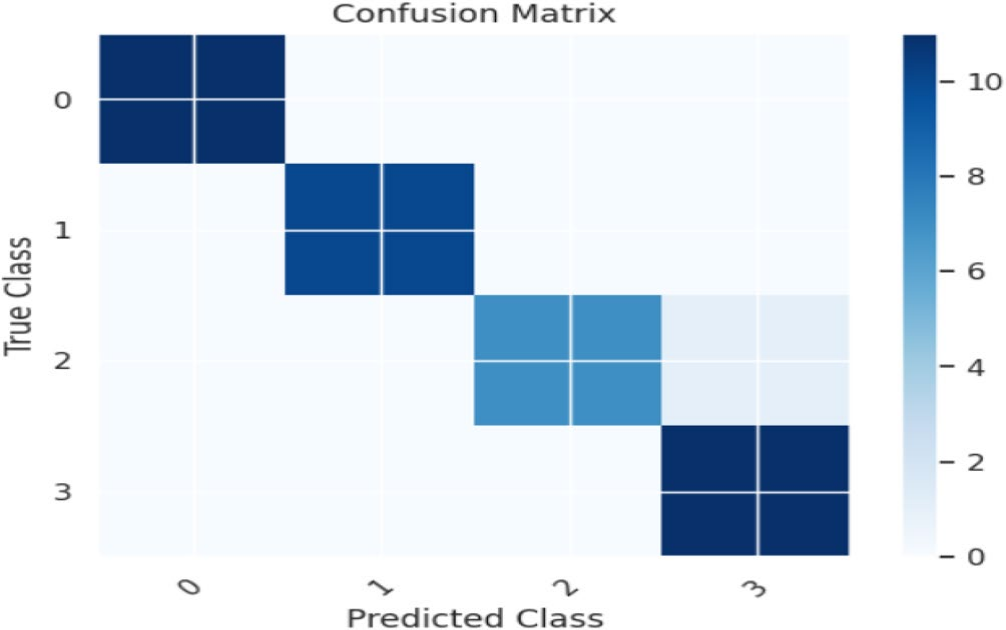
KNN classifies confusion matrix containing predicted class and true class.

**Figure 13:**
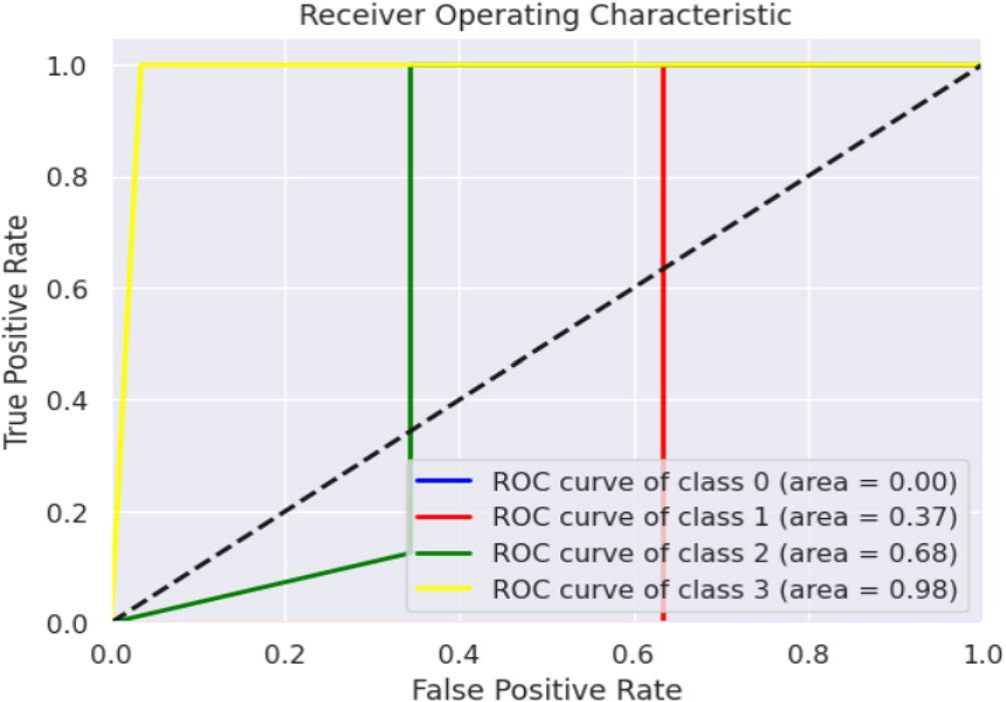
Performance Characteristics of KNN classifier.

Evaluation metrics, such as precision, accuracy, specificity, F1-score, and sensitivity, are estimated. The K-Nearest Neighbour Classifier's confusion matrix between the predicted and actual classes is displayed in Fig. 12. Figure 13 shows the overall performance Characteristics of KNN.

**Figure 14:**
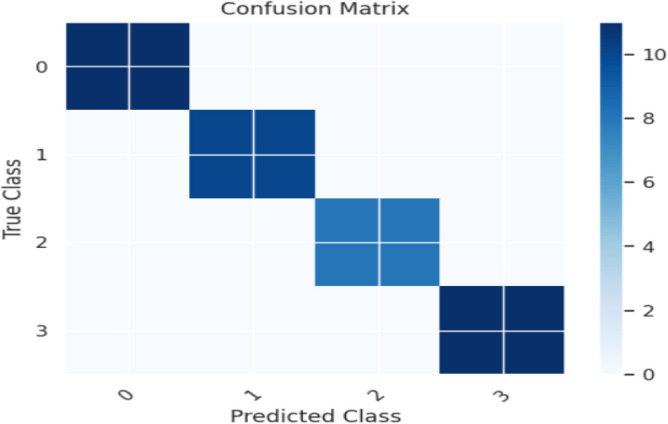
Random Forest classifier confusion matrix containing predicted class and true class.

**Figure 15:**
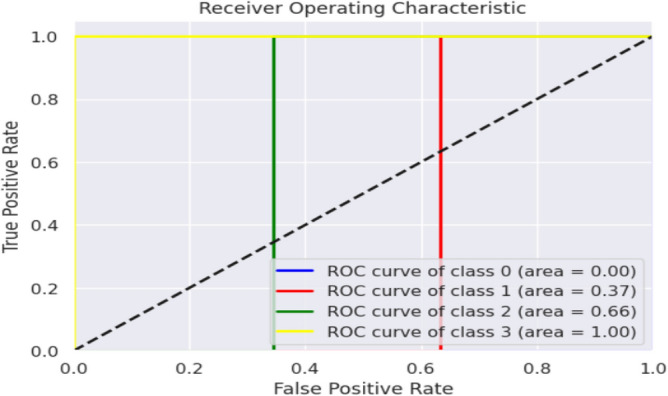
Performance Characteristics of Random Forest classifier.

The Random Forest Classifier's confusion matrix between the predicted and actual classes is displayed in Fig. 14. Figure 15 shows the overall performance Characteristics of Random Forest Classifier.

**Figure 16:**
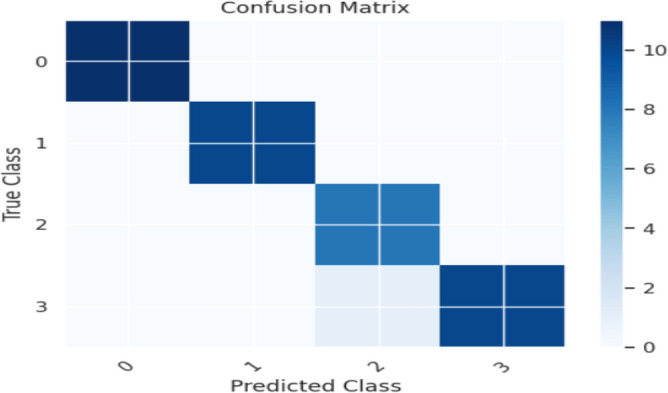
CATBOOST classifier confusion matrix containing predicted class and true class.

**Figure 17:**
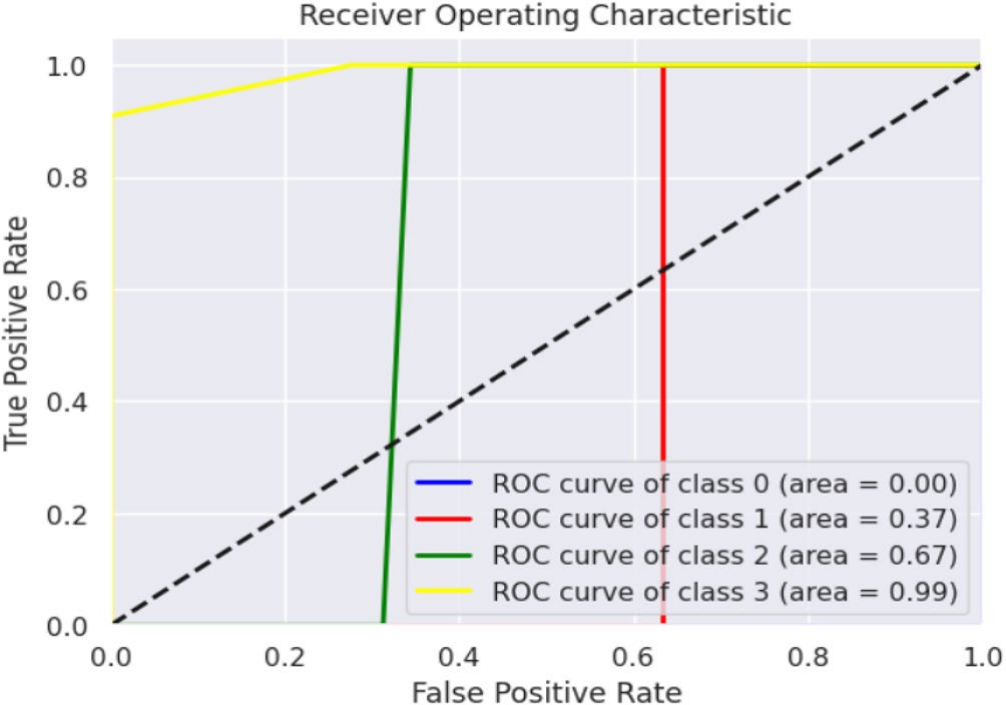
Performance Characteristics of CATBOOST classifier.

The CATBOOST Classifier's confusion matrix between the predicted and actual classes is displayed in Fig. 16. Figure 17 shows the overall performance Characteristics of CATBOOST Classifier.

**Figure 18:**
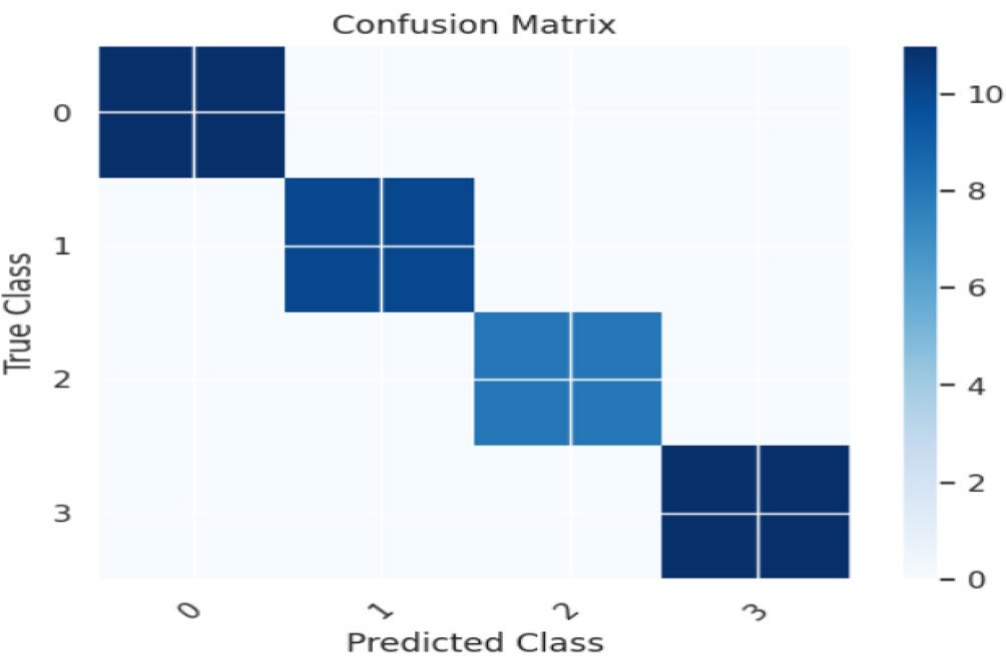
XGBOOST classifier confusion matrix containing predicted class and true class.

**Figure 19:**
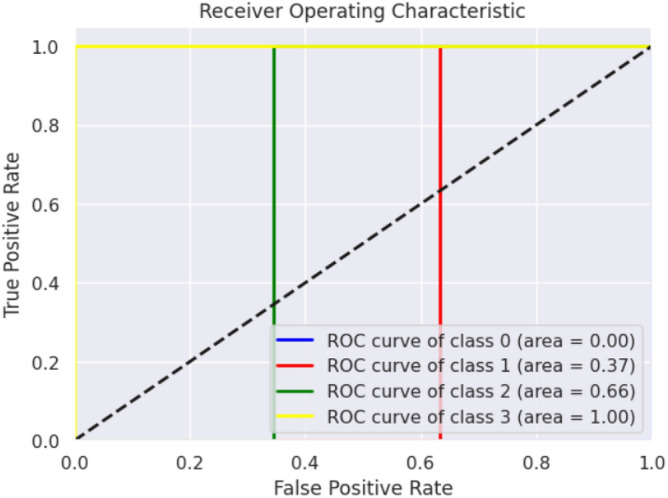
Performance Characteristics of XGBOOST classifier.

The XGBOOST Classifier's confusion matrix between the predicted and actual classes is displayed in Fig. 18. Figure 19 shows the overall performance Characteristics of XGBOOST Classifier.

The performance parameters used for the analysis using four different classifiers are given with mathematical equations. Accuracy helps to predict the outcome of the model correctly. Precision metric helps to depict the model ability to measure the accuracy of positive predictions only. Sensitivity or Recall is the capability of the model to depict all positive states from actual positive states. Specificity is the ability of the model to depict all negative states from actual negative states. F1 score depict the harmony between precision and recall. Its higher value indicates how a model provides good balance by minimizing false negatives and false positives.

1. Accuracy = $$\frac{{\text{TP + TN}}}{{\text{TP + FP + FN + TN}}}$$

2. Precision = $$\frac{{{\text{TP}}}}{{\text{TP + FP}}}$$

3. Sensitivity = $$\frac{{{\text{TP}}}}{{\text{TP + FN}}}$$

4. Specificity = $$\frac{{{\text{TN}}}}{{\text{TN + FN}}}$$

5. F1-score = $$\frac{{{2}\,{*}\,\,{\text{precision}}\,{\text{x}}\,\,{\text{Recall}}}}{{{\text{Precision}}\,\,{ + }\,{\text{Recall}}}}$$

Where, Rwcall = $\frac{{{\text{TP}}}}{{\text{TP = + FN}}}$ Precision = $\frac{{{\text{TP}}}}{{\text{TP + FP}}}$

TP are true positive states, FP are False positive states, TN are true Negative states and FN are False Negative states.

The performance parameters by using KNN, RF, CATBOOST and XGBOOST are calculated with these equations as shown in Table 4.

**Table 4:** Evaluation Parameters for all the classifiers.

Parameters	Accuracy	Precision	Recall	F1 Score
KNN	0.975	0.977	0.975	0.974
RF	1	1	1	1
CATBOOST	0.975	0.978	0.975	0.975
XGBOOST	1	1	1	1

The comparative analysis as depicted from Table 4 shows that amongst all the four classifiers we achieved 100% accuracy in random forest and XGBOOST classifier.

## Conclusion

In conclusion, detecting food adulteration, such as oil adulteration, is an important task for ensuring food safety and quality. Random forest and XGBOOST are algorithms that can be trained on a refractive index dataset and real time database to make predictions on new samples. By creating a PyQT5 application, the random forest model can be incorporated into a user-friendly interface for predicting the refractive index of new oil samples as well as real time data samples and detecting adulteration. In the proposed work mixture of edible oil with palm oil is taken into consideration for both the approaches. In future the work can be extended to the mixture of edible oil with animal fats for refractive index as well as fumigation method approaches. The method presented in this study helps to tackle the issue of detecting food adulteration, which has broad societal advantages.

## Data Availability

The datasets used during this work are available from the corresponding author on reasonable request.
